# Enterococcus faecalis Aortic Valve Endocarditis Masquerading As Urinary Tract Infection: Diagnostic Delay, Septic Embolic Strokes, and a Fatal Therapeutic Impasse

**DOI:** 10.7759/cureus.114387

**Published:** 2026-08-11

**Authors:** Muhammad A Tariq, Sara Tariq, Ayesha Munir, Syed Hamza Waheed, Shannay E Bellamy

**Affiliations:** 1 Internal Medicine, Guthrie Lourdes Hospital, Binghamton, USA; 2 Medicine and Surgery, Quaid-e-Azam Medical University, Bahawalpur, PAK; 3 Internal Medicine, Rochester Regional Health, Rochester, USA; 4 Internal Medicine, Jersey City Medical Center, Jersey City, USA

**Keywords:** aortic valve vegetation, enterococcus faecalis, hemorrhagic transformation, infective endocarditis, perivalvular abscess, septic emboli, transesophageal echocardiography

## Abstract

We present a case involving a 67-year-old man with multiple comorbidities who initially experienced two weeks of gastrointestinal symptoms that were managed as acute gastroenteritis. He later returned to the emergency department with altered mental status following a fall. Initial evaluation showed leukocytosis, findings consistent with a urinary tract infection, and elevated cardiac biomarkers. Head computed tomography revealed a right frontal lobe hypodensity, while imaging of the chest, abdomen, and pelvis showed diffuse lymphadenopathy. Blood cultures identified vancomycin-susceptible *Enterococcus faecalis*, and urine cultures grew *Escherichia coli*, indicating two separate infections. Although transthoracic echocardiography was initially negative, transesophageal echocardiography (TEE) later confirmed aortic valve endocarditis with a perivalvular abscess and a possible left atrial appendage thrombus. The patient’s course was complicated by multifocal embolic cerebral infarctions with hemorrhagic transformation, subarachnoid hemorrhage, new-onset atrial fibrillation, acute hypoxic respiratory failure due to volume overload, and acute kidney injury. He was considered a prohibitively high-risk surgical candidate because of recent cerebrovascular events and extensive comorbidities. After a prolonged hospitalization involving multiple facility transfers and multidisciplinary evaluation, the patient chose comfort measures and subsequently died. This case underscores the diagnostic challenges of infective endocarditis presenting with nonspecific symptoms, the value of TEE when clinical suspicion remains despite a negative transthoracic study, and the severe consequences of delayed diagnosis.

## Introduction

Infective endocarditis (IE) remains a diagnostic and therapeutic challenge, causing significant morbidity and mortality despite advances in imaging and antimicrobial therapy [1]. The global burden of IE has increased sharply, with incident cases rising from approximately 478,000 in 1990 to over 1,090,000 in 2019. The age-standardized incidence also increased from 9.9 to 13.8 per 100,000 person-years during this period [1]. This rise is largely driven by an aging population. For example, a Scotland-wide study found that the incidence rate among patients aged 80 years or older doubled between 1990 and 2014. Enterococcal bacteremia is associated with the second-highest one-year mortality among IE pathogens (adjusted odds ratio (OR), 3.41; 95% confidence interval (CI), 2.04-5.70), exceeded only by *Staphylococcus aureus* [2]. In-hospital mortality remains high, occurring in 17.1% of patients in the European Society of Cardiology-EURObservational Research Programme-European Endocarditis Registry (ESC-EORP EURO-ENDO), the largest contemporary prospective IE cohort (n=3,116). Independent predictors of mortality include cerebral complications, perivalvular abscess, congestive heart failure, and failure to perform surgery when indicated [1].

*Enterococcus faecalis* is the third most common cause of IE, accounting for approximately 10% of cases, and is increasingly recognized in elderly patients with comorbidities and healthcare exposures [1]. The classic presentation of IE, fever, a new murmur, and embolic phenomena, is often absent, especially in older adults, who may present with nonspecific symptoms such as malaise, confusion, and weight loss [1]. In the EURO-ENDO registry, fever was present in 77.7% of patients and cardiac murmurs in 64.5%, but classic peripheral stigmata were rare: Janeway lesions in 3.5%, Osler nodes in 1.9%, and Roth spots in 1.4% [1]. This atypical presentation often leads to diagnostic delays, which are associated with higher rates of complications, including embolic stroke, perivalvular abscess, and heart failure [1]. Embolic events occurred in 20.6% of patients in the EURO-ENDO cohort and were significantly associated with vegetations and *S. aureus* infection [1]. Up to 30% of patients with subsequently confirmed IE are initially classified as having “possible” IE because of equivocal or negative echocardiographic or blood culture findings, highlighting the diagnostic challenges of this disease [3].

We present this case to highlight three underappreciated clinical challenges: (1) the diagnostic pitfall of discordant blood and urine culture organisms that can delay recognition of endocarditis, (2) the inadequacy of transthoracic echocardiography (TTE) alone in obese patients with high clinical suspicion for IE, and (3) the therapeutic impasse that arises when surgical indications and neurologic contraindications coexist. These lessons are particularly relevant given the rising incidence of enterococcal IE in elderly patients with comorbidities.

## Case presentation

Initial presentation and outpatient encounter

In October 2025, a 67-year-old man with a past medical history of insulin-dependent type 2 diabetes mellitus, diabetic neuropathy, morbid obesity (weight approximately 440 lb), hypertension, bilateral knee osteoarthritis, lower-extremity stasis ulcers, and bilateral hydrocele presented to an outpatient clinic with a two-week history of diarrhea, nausea, weakness, and subjective fever. Social history was notable for a sedentary lifestyle; he denied tobacco, alcohol, or illicit drug use, including intravenous drug use. Family history was noncontributory. He was diagnosed with acute gastroenteritis and managed conservatively with oral rehydration, ondansetron 4 mg orally as needed, and famotidine 20 mg orally twice daily.

Emergency department presentation (Day 1)

The patient was brought to the emergency department (ED) by emergency medical services (EMS) after being found at home on the floor, soaked in urine, and altered following a fall. Vital signs were notable for tachypnea (respiratory rate, 33 breaths/min), oxygen saturation of 95% on 3 L/min via nasal cannula, afebrile status, and stable blood pressure.

On examination, the patient appeared lethargic and was oriented only to self. Cardiac auscultation revealed a regular rate and rhythm without appreciable murmurs, rubs, or gallops, though auscultation was limited by body habitus. Lungs were clear to auscultation bilaterally. The abdomen was soft, nontender, and obese. The lower extremities demonstrated bilateral chronic stasis ulcers with surrounding hyperpigmentation and 1+ pitting edema. No Janeway lesions, Osler nodes, splinter hemorrhages, or conjunctival petechiae were identified. No track marks or signs of intravenous drug use were observed.

Key laboratory findings are summarized in Table 1.

**Table 1:** Initial laboratory values at ED presentation (Day 1) WBC: white blood cell; RBC: red blood cell; CPK: creatine phosphokinase; ALT: alanine transaminase; ED: emergency department

Parameter	Result	Reference range
WBC (×10³/µL)	17.21	4.5-11.0
Hemoglobin (g/dL)	11.9	13.5-17.5
Platelets (×10³/µL)	143	150-400
Sodium (mEq/L)	135	136-145
Glucose (mg/dL)	279	70-100
Troponin (ng/mL)	2.29	0.04
CPK (U/L)	295	22-198
Total bilirubin (mg/dL)	1.2	0.1-1.2
ALT (U/L)	42	7-56
Urine leukocytes	215	Negative
Urine bacteria	4+	Negative
Urine WBC	High	0-5/HPF
Urine RBC	High	0-3/HPF

**Figure 1:**
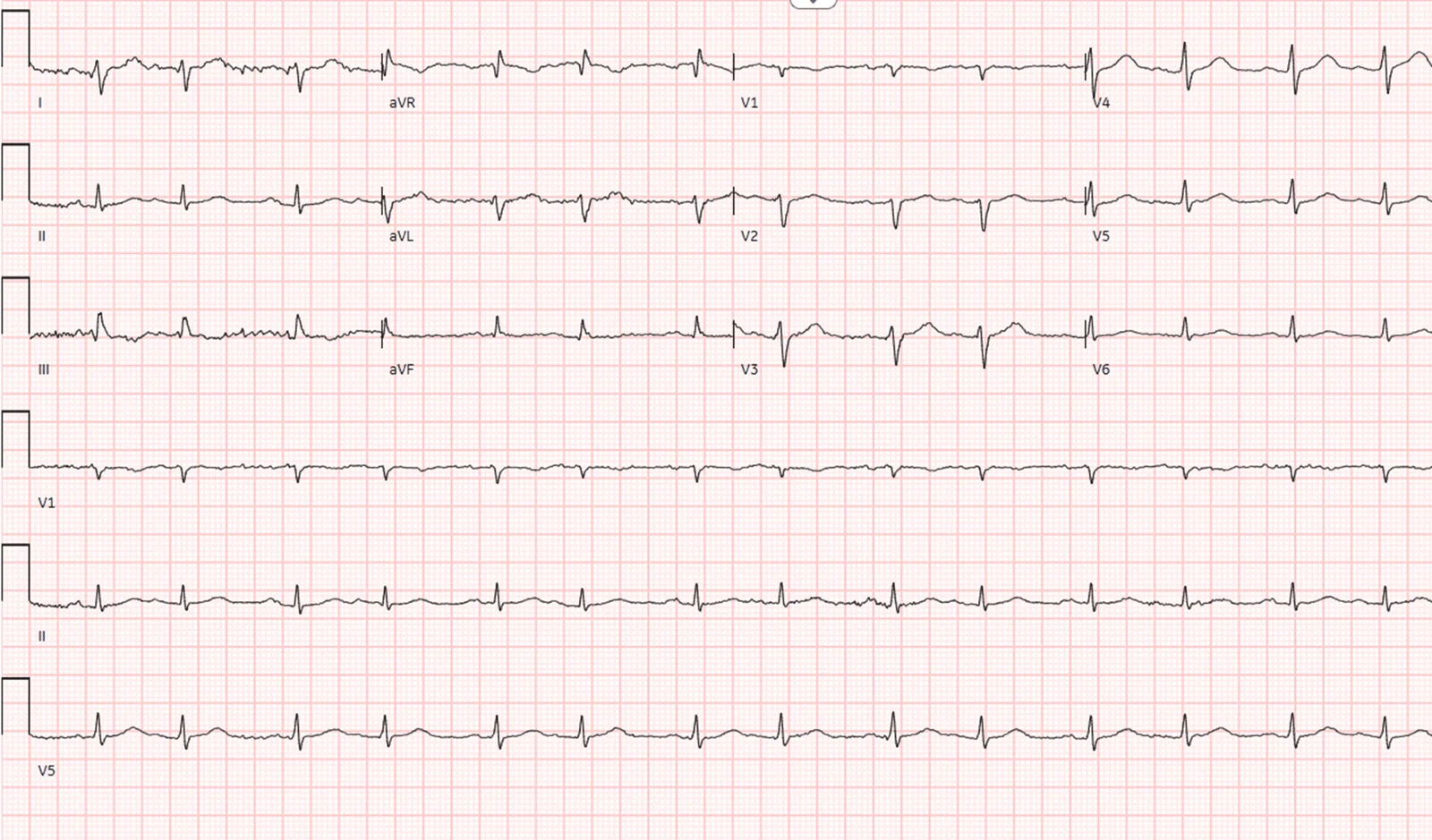
ECG showing sinus rhythm at a VR of 85 beats/min, without ischemic changes ECG: electrocardiography, VR: ventricular rate

Notable results included leukocytosis, elevated troponin, and thrombocytopenia. Urinalysis revealed leukocytes and 4+ bacteria. ECG showed sinus rhythm without ischemic changes (Figure 1).

**Figure 2:**
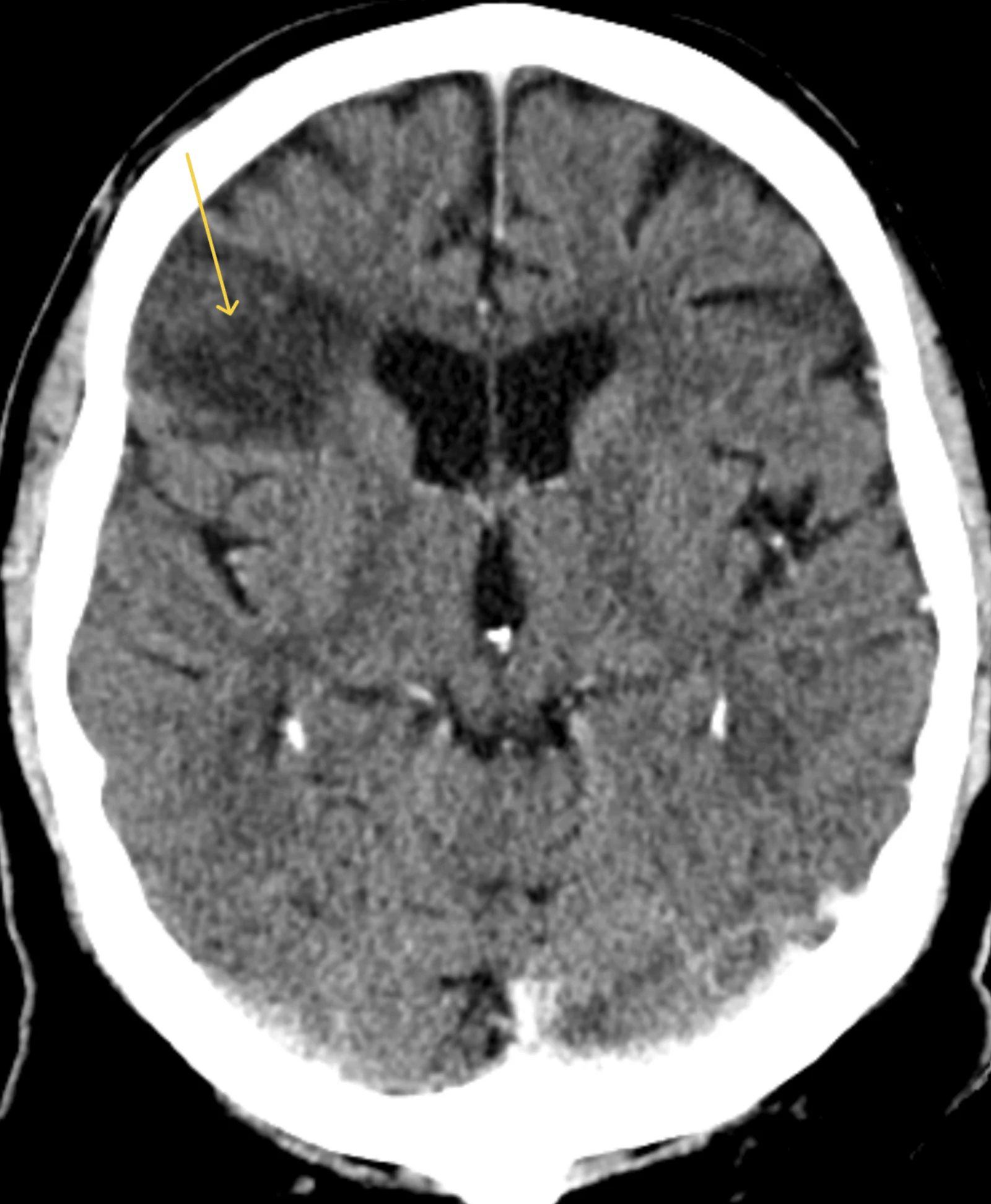
Axial non-contrast CT of the head on Day 1 demonstrating an approximately 3 cm focus of hypoattenuation in the right frontal lobe (yellow arrow), initially interpreted as an infarction of indeterminate age versus a neoplastic lesion CT: computed tomography

Computed tomography (CT) head without contrast revealed a 3 cm hypodense focus in the right frontal lobe, interpreted as indeterminate for age infarction versus neoplastic lesion (Figure 2).

**Figure 3:**
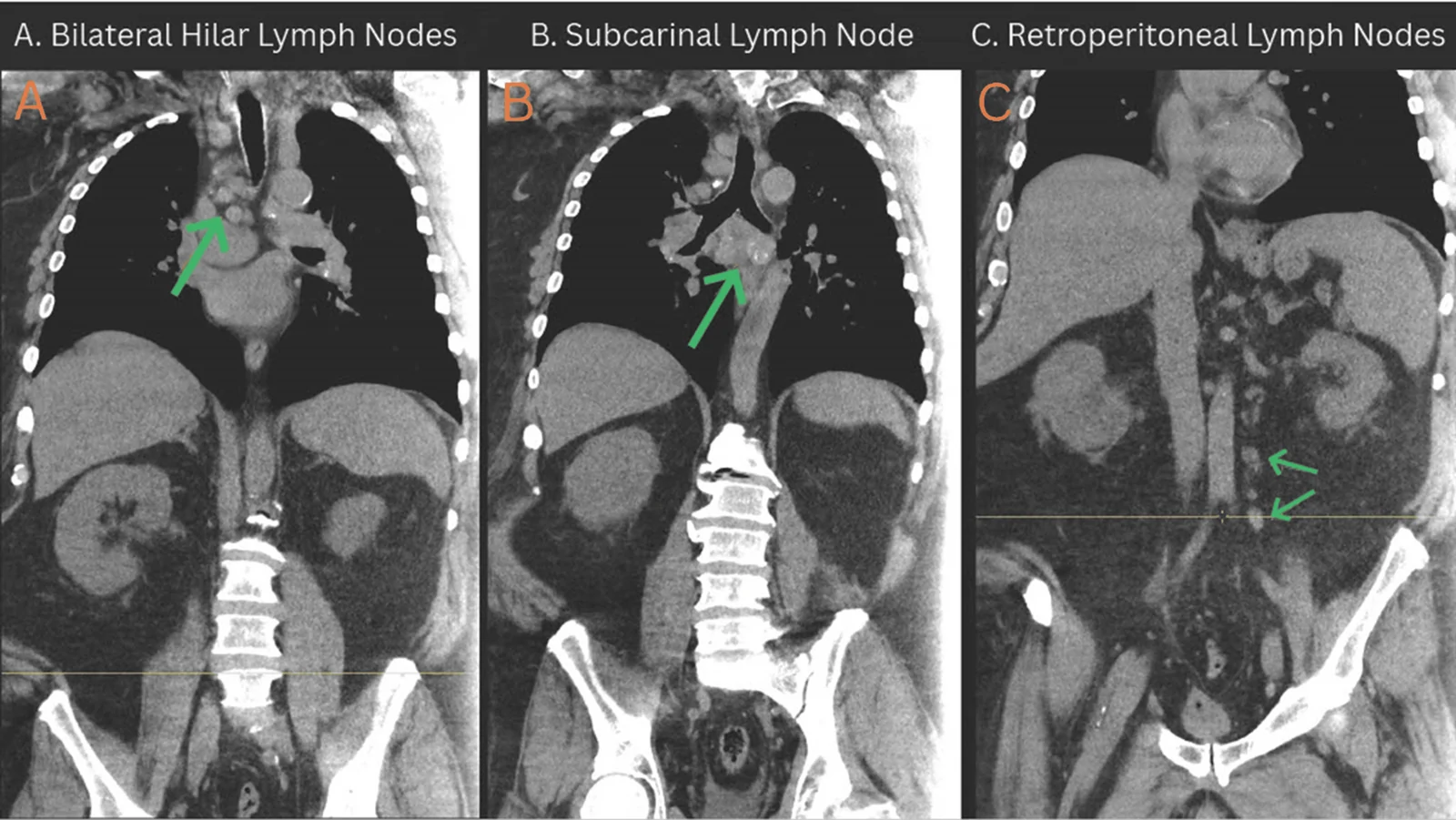
Contrast-enhanced coronal CT images demonstrating lymphadenopathy in multiple nodal stations: (A) bilateral hilar lymphadenopathy (arrow), (B) enlarged subcarinal lymph node (arrow), and (C) retroperitoneal lymphadenopathy in the para-aortic region (arrows) CT: computed tomography

CT of the chest, abdomen, and pelvis showed diffuse lymphadenopathy involving bilateral hilar, mediastinal, subcarinal, retroperitoneal, and inguinal lymph nodes (Figure 3). Findings were suspicious for neoplastic involvement. Hepatomegaly and fatty infiltration were present, but no primary mass was identified.

The patient received an intravenous bolus of lactated Ringer’s solution, intravenous ceftriaxone 2 g once daily, intravenous dexamethasone 10 mg once, and an oral loading dose of aspirin 325 mg. He was admitted for sepsis secondary to a presumed urinary tract infection (UTI).

Hospital Days 2-5: divergent cultures, evolving neuroimaging, and the search for a source

Blood cultures grew vancomycin-susceptible *E. faecalis*, while the urine culture grew *Escherichia coli*, confirming two distinct organisms and indicating that the UTI was not the source of bacteremia. Intravenous vancomycin (dosed to achieve trough levels of 15-20 µg/mL, with an initial trough of 17.5 µg/mL) was subsequently added to ceftriaxone. On Day 2, an initial TTE showed a preserved ejection fraction of 55%-60% with no wall motion abnormalities or vegetations; however, image quality was significantly limited by the patient's body habitus, precluding definitive exclusion of valvular pathology (Video 1).

**Video 1:** Transthoracic echocardiogram (parasternal short-axis view of the aortic valve) demonstrating a preserved left ventricular ejection fraction of 55%-60% without wall motion abnormalities or clearly visualized vegetations. Image quality was significantly limited due to the patient’s body habitus, resulting in poor acoustic windows. The aortic valve leaflets were not well visualized, and although no obvious vegetations were identified, the study was technically limited and could not definitively exclude valvular pathology

**Figure 4:**
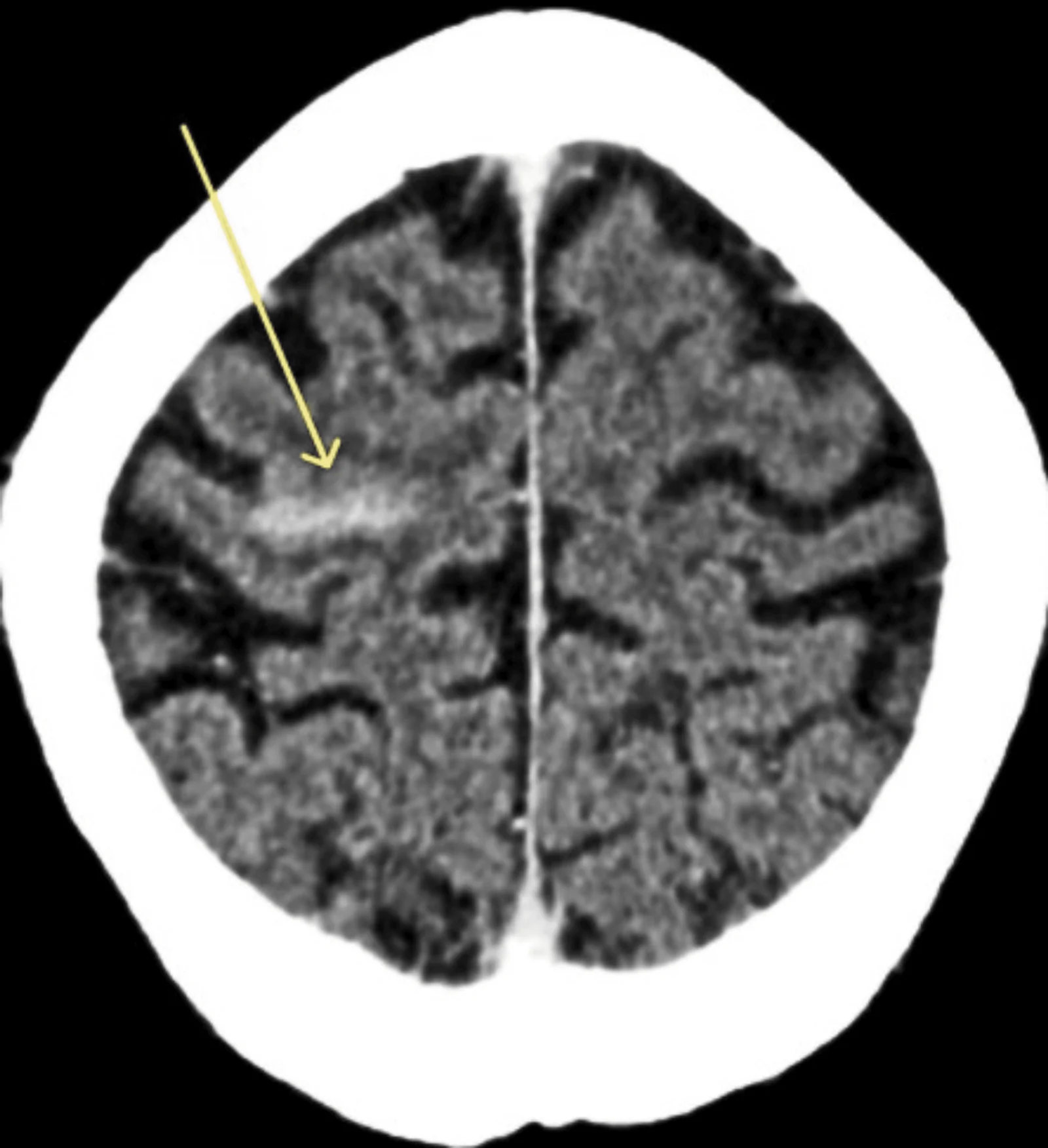
Axial contrast-enhanced CT of the head on Day 3 demonstrating acute right frontal SAH (yellow arrow), with unchanged right frontal lobe hypoattenuation consistent with a chronic infarct CT: computed tomography; SAH: subarachnoid hemorrhage

CT head with contrast on Day 3 revealed an acute right frontal subarachnoid hemorrhage (SAH) (Figure 4).

**Figure 5:**
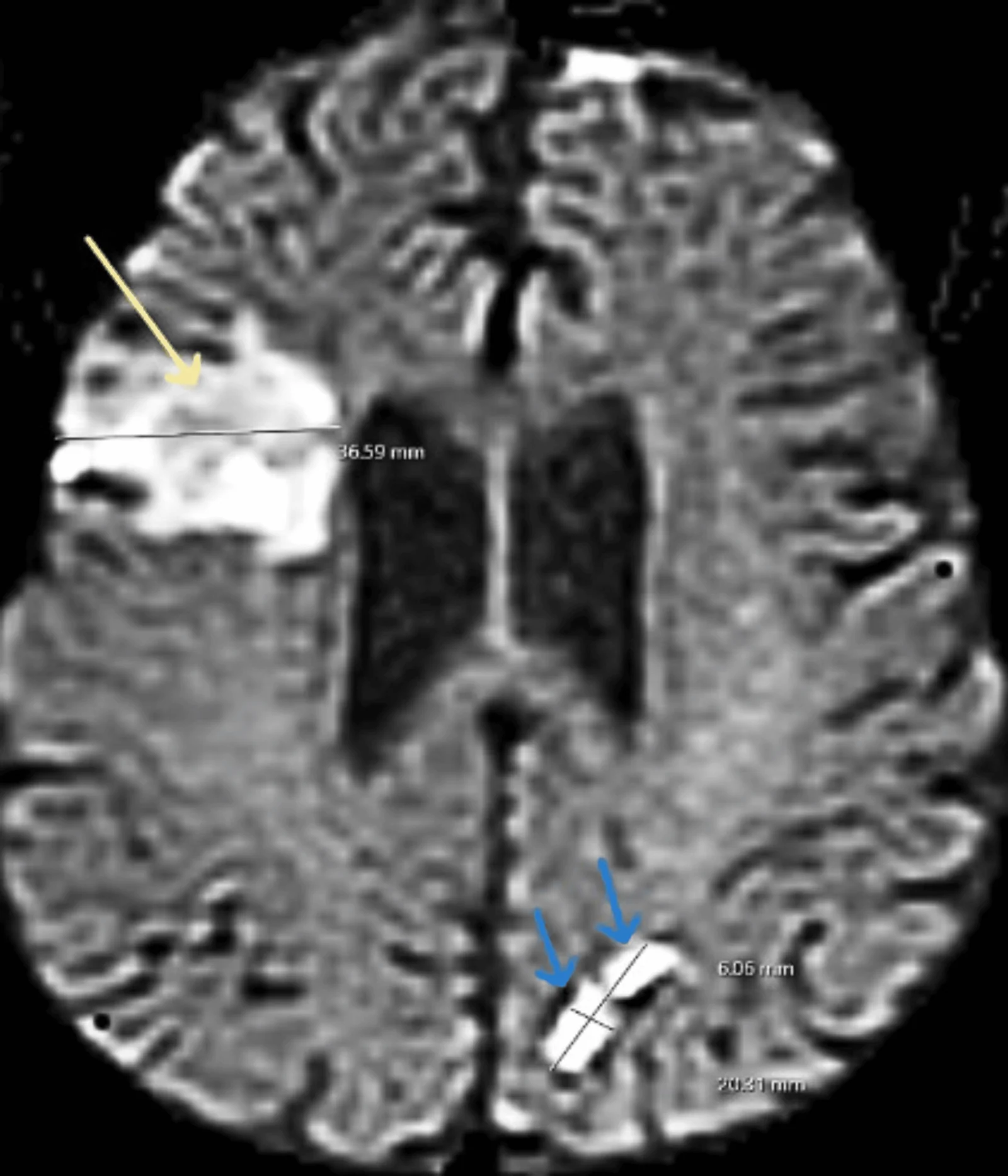
MRI of the brain on Day 4. DWI demonstrating restricted diffusion in the right frontal lobe consistent with a 36.59 mm subacute infarction (yellow arrow) and additional subacute infarctions in the left parietal lobe (blue arrows), confirming a bilateral embolic distribution MRI: magnetic resonance imaging; DWI: diffusion-weighted imaging

**Figure 6:**
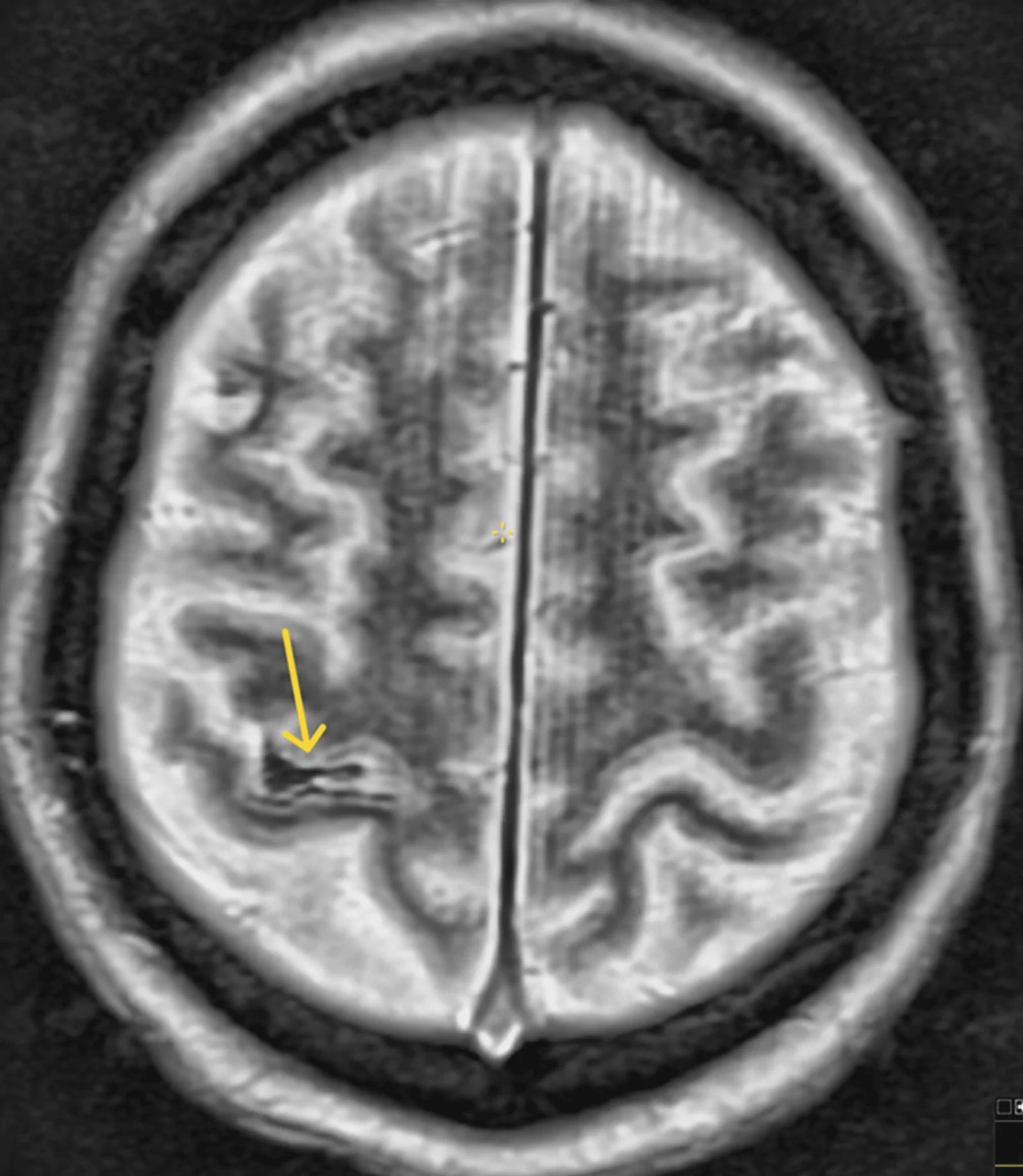
MRI of the brain on Day 4, with GRE sequence demonstrating a curvilinear band of hemorrhagic products in the high right parietal lobe (yellow arrow), compatible with hemorrhagic conversion of a subacute infarction MRI: magnetic resonance imaging; GRE: gradient-echo

On hospital Day 3, oral nimodipine 60 mg every four hours was started for vasospasm prophylaxis, blood pressure parameters were tightened, and one unit of platelets was transfused. MRI of the brain performed on hospital Day 4 demonstrated multifocal subacute infarctions, a 3.6 cm right frontal infarct, smaller infarcts in the high right frontal and left parietal lobes, and hemorrhagic conversion in the right parietal lobe (Figures 5, 6). The bilateral distribution strongly suggested a proximal embolic source, most likely cardioembolic given the concurrent *E. faecalis* bacteremia and absence of significant large-artery stenosis.

The bubble study was negative for a right-to-left shunt (Video 2).

**Video 2:** Apical four-chamber view demonstrating a negative bubble study to assess for an intracardiac shunt

**Figure 7:**
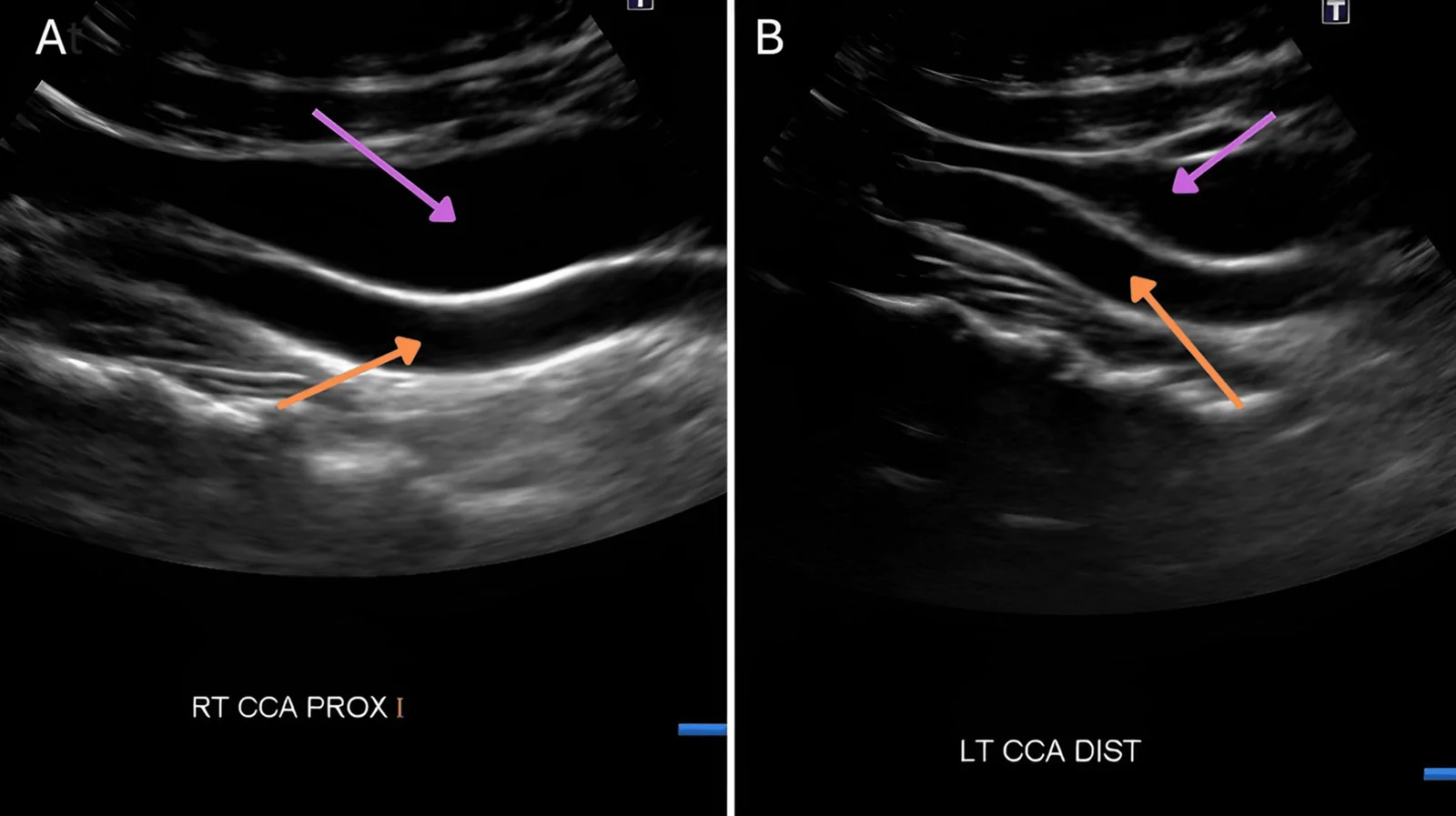
Gray-scale ultrasound images of the right proximal (A) and left distal (B) common carotid arteries demonstrating mild (<50%) stenosis with preserved luminal patency. The common carotid artery (orange arrows) and adjacent internal jugular vein (purple arrows) are labeled for orientation RT: right; LT: left; CCA: common carotid artery; PROX: proximal; DIST: distal

Carotid duplex showed less than 50% stenosis bilaterally (Figure 7).

**Figure 8:**
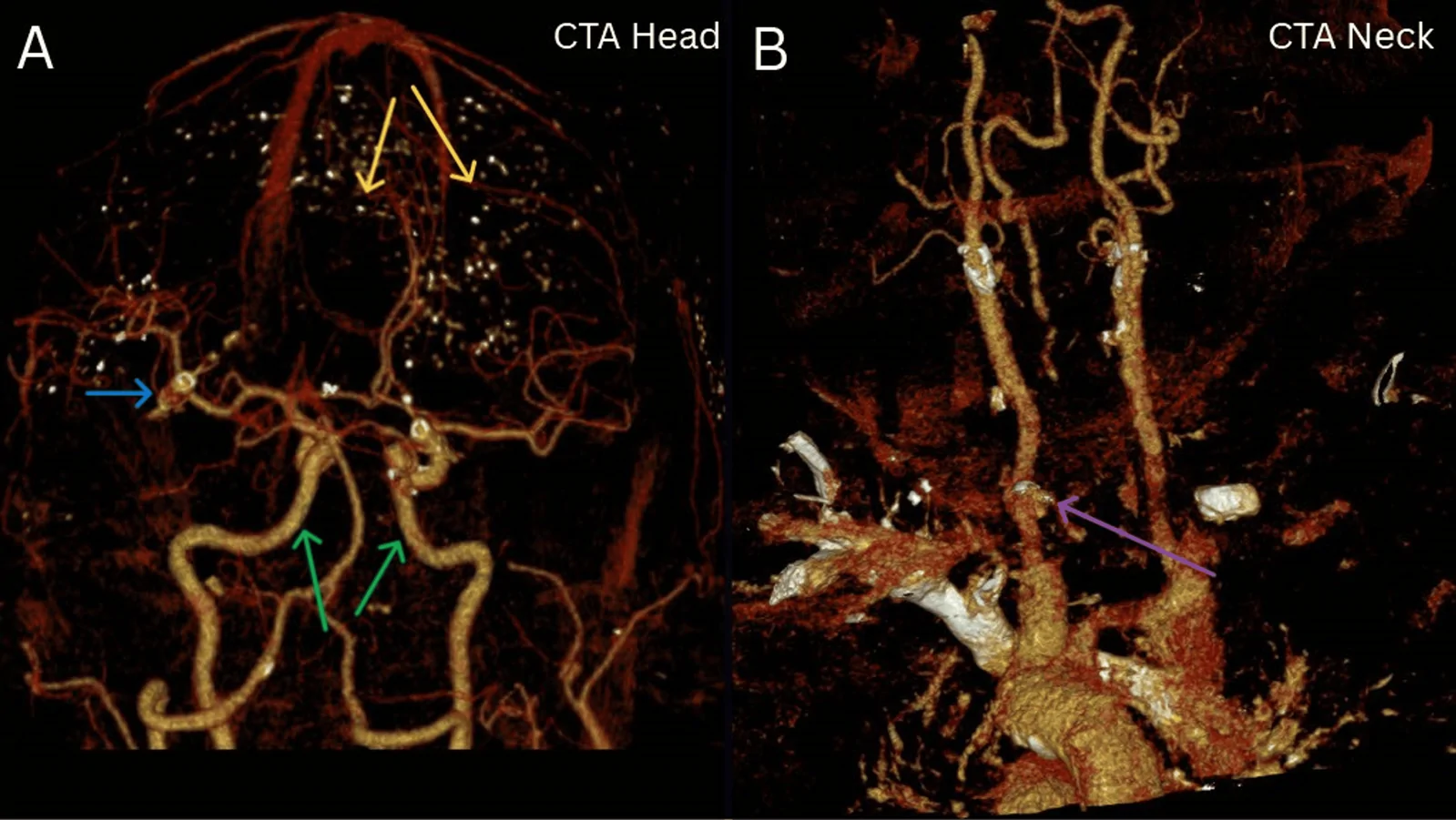
Three-dimensional volume-rendered CTA images of the head (A) and neck (B) demonstrate normal opacification and course of the major arterial vasculature. In A, the intracranial circulation is visualized, including the anterior cerebral arteries (yellow arrows), middle cerebral arteries (blue arrow), and internal carotid arteries (green arrows). In B, the cervical vasculature shows the common carotid arteries (purple arrow) with normal caliber and bifurcation. There is no evidence of significant stenosis, aneurysm, dissection, or vascular malformation CTA: computed tomography angiography

Computed tomography angiography (CTA) of the head and neck demonstrated no significant stenoses or aneurysms (Figure 8).

Aspirin was discontinued on hospital Day 4. Repeat blood cultures at 48 hours remained positive for *E. faecalis* (Table 2). Neurology and infectious diseases (ID) were consulted; neurology suspected a cardioembolic etiology and recommended transesophageal echocardiography (TEE), while ID recommended daily blood cultures until clearance.

**Table 2:** Blood and urine culture timeline

Day	Specimen	Organism	Susceptibility	Antibiotic regimen
1	Blood	Enterococcus faecalis	Vancomycin-susceptible	Ceftriaxone
1	Urine	Escherichia coli	Pan-sensitive	Ceftriaxone
3	Blood	E. faecalis	Vancomycin-susceptible	Ceftriaxone+vancomycin
5	Blood	E. faecalis	Vancomycin-susceptible	Ceftriaxone+vancomycin
6	Blood	E. faecalis	Vancomycin-susceptible	Ceftriaxone+vancomycin
7	Blood	E. faecalis	Vancomycin-susceptible	Ceftriaxone+vancomycin
9	Blood	No growth (48 hrs)	-	Vancomycin
-	Blood	No growth	-	Ampicillin+ceftriaxone

Hospital Day 6: the diagnosis

**Figure 9:**
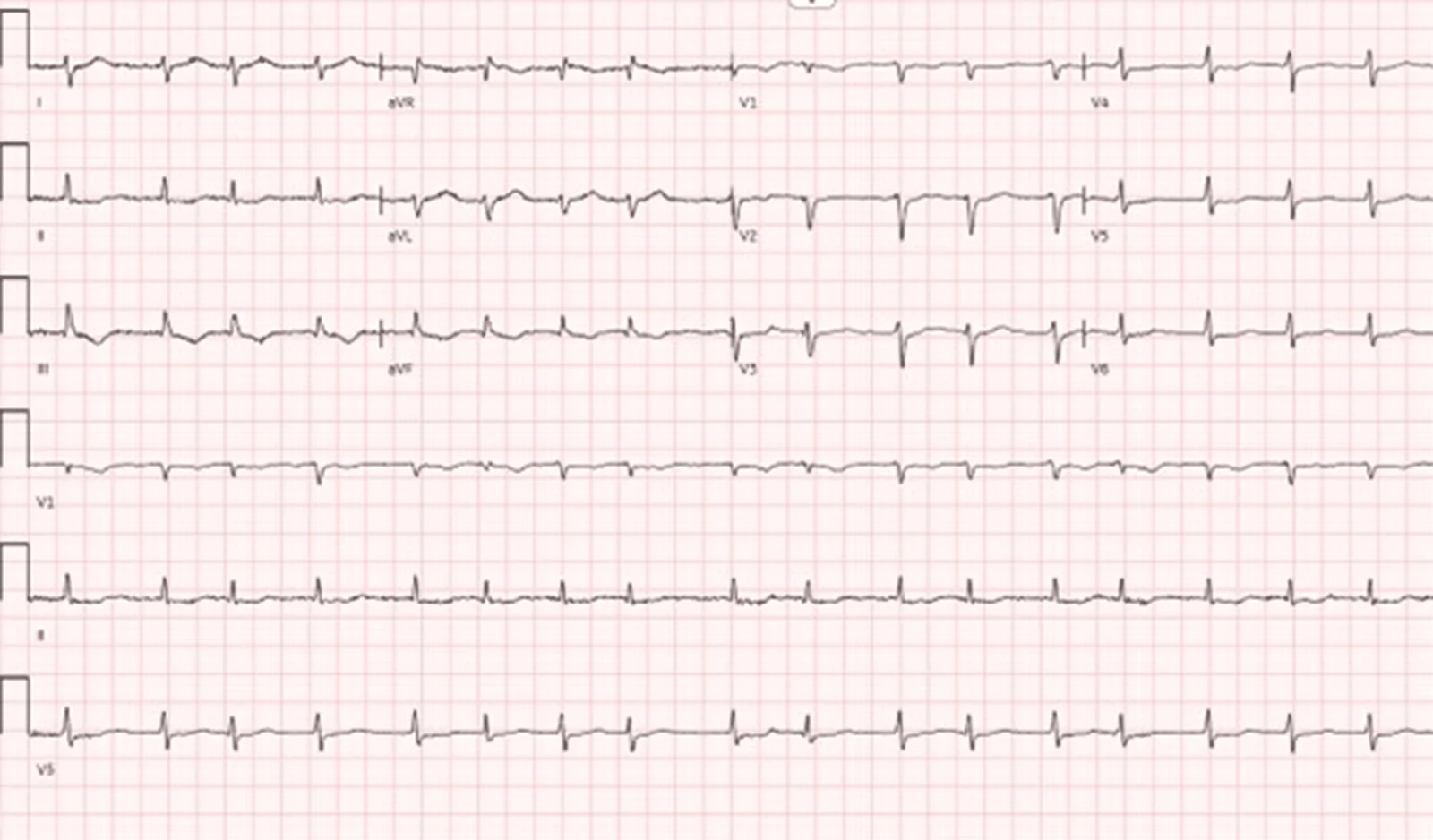
Twelve-lead ECG on Day 6 demonstrating new-onset atrial fibrillation with a ventricular rate of 97 beats per minute ECG: electrocardiography

The patient developed new-onset atrial fibrillation (heart rate, 100-120 beats/min; asymptomatic); intravenous metoprolol 5 mg was administered for acute rate control, followed by oral metoprolol tartrate 12.5 mg twice daily for maintenance (Figure 9).

**Figure 10:**
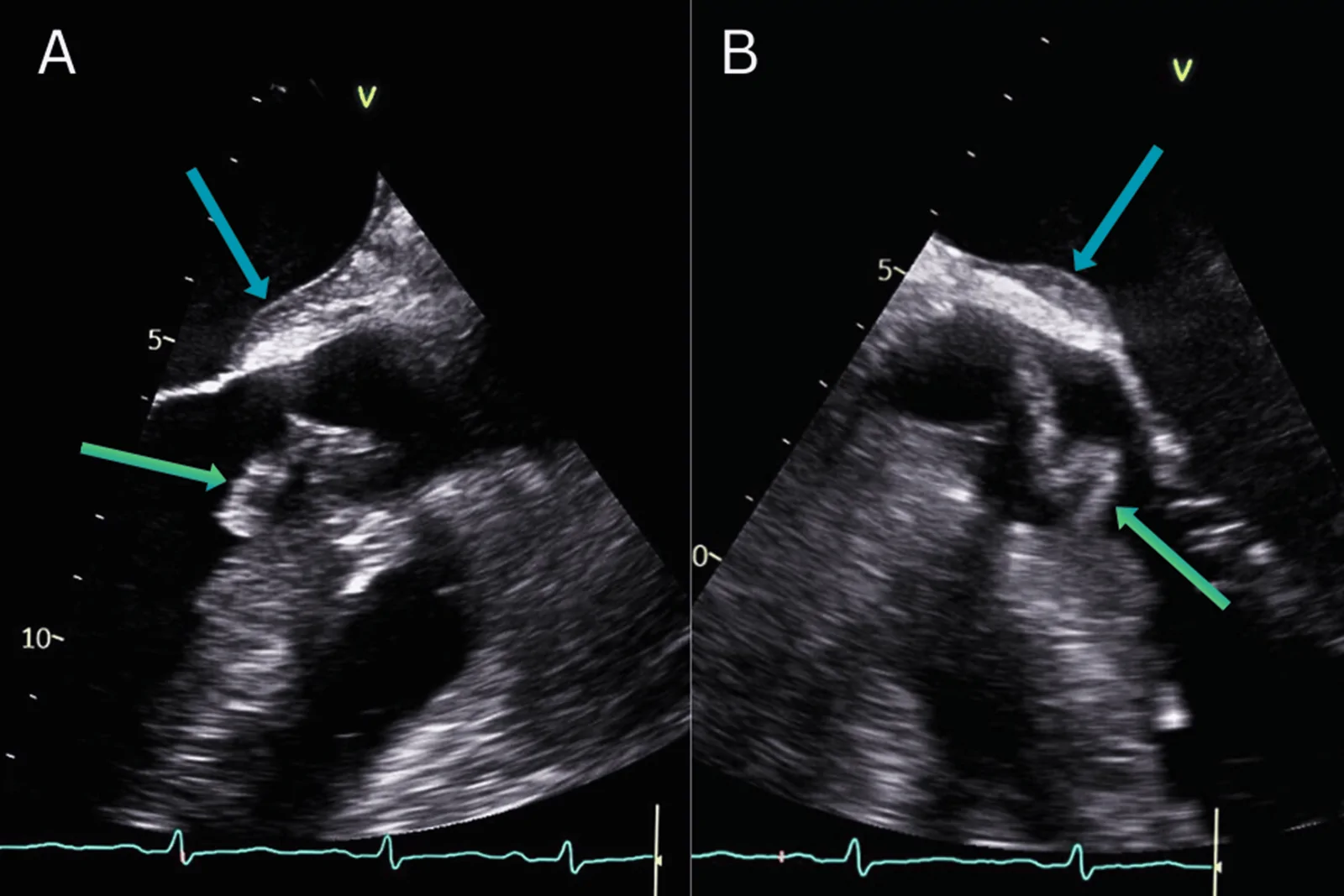
Transesophageal echocardiogram: (A) mid-esophageal view (130°) and (B) mid-esophageal view (0°) demonstrating a heterogeneous echolucent appearance of the aortic root, suggesting a paravalvular abscess (blue arrows) and vegetation on the cusp of the aortic valve (green arrows)

Thyroid-stimulating hormone (TSH) was elevated at 9.12 µIU/mL (reference range, 0.27-4.20 µIU/mL). TEE revealed aortic valve endocarditis with a possible left atrial appendage (LAA) thrombus, findings missed on TTE (Figure 10).

Blood cultures from hospital Days 5-7 continued to grow *E. faecalis*, representing persistent bacteremia despite appropriate therapy (Table 2). Following confirmation of IE, a planned six-week course of intravenous antibiotic therapy was initiated.

Surgical evaluation across three facilities (Days 7-25)

The unified diagnosis was *E. faecalis* aortic valve endocarditis with a perivalvular abscess, persistent bacteremia, bilateral septic embolic strokes with hemorrhagic transformation, SAH, new-onset atrial fibrillation, and LAA thrombus. The patient was transferred twice for escalating levels of cardiothoracic surgical evaluation (Table 3).

**Table 3:** Surgical risk assessment across facilities STS: Society of Thoracic Surgeons; CT: computed tomography; SAH: subarachnoid hemorrhage

Facility	Day	STS risk score	6-month mortality	Decision	Rationale
Community hospital	1-7	Not calculated	-	Transfer for surgical evaluation	No CT surgery capability; perivalvular abscess and SAH
Regional medical center	8-10	10%	67%	Transfer to higher-level facility	Elevated risk, evolving head CT, comorbidities
Quaternary care center	12-25+	-	-	Defer surgery ≥1 month	Stable hemodynamics; stroke/SAH risk with anticoagulation

At the first receiving facility (Day 8), CT surgery calculated a Society of Thoracic Surgeons (STS) risk score of 10% and a six-month mortality risk of 67%, deeming him not a surgical candidate given the evolving head CT findings. Blood cultures cleared on Day 9. At the quaternary care center (Day 12 onward), digital subtraction angiography (DSA) was negative for mycotic aneurysms. A repeat TEE (Day 20) showed severe aortic regurgitation from a flail left coronary cusp but notably no LAA thrombus or perivalvular abscess, differing from the initial TEE.

Neurology assessed the anticoagulation risk as acceptable but recommended deferring surgery for one month if the patient remained hemodynamically stable. Cardiac surgery concurred; the patient did not require urgent intervention.

After a palliative care consultation, the patient elected medical management without surgical intervention. In discussions with the palliative care team, the patient expressed that he did not wish to pursue further invasive interventions and prioritized comfort and time with his family over aggressive surgical management.

Decompensation and death

On hospital Day 26, the patient developed acute hypoxic respiratory failure due to volume overload and acute kidney injury (AKI), with creatinine rising from a baseline of 0.8 to 1.8 mg/dL, requiring transfer to the cardiac intensive care unit (CICU) for intravenous bumetanide continuous infusion at 4 mg/hour and bilevel positive airway pressure (BiPAP) (Table 4).

**Table 4:** Laboratory trends across hospitalization ^*^Post-transfusion of 1 unit of platelets TSH: thyroid-stimulating hormone; WBC: white blood cell

Parameter	Day 1	Day 3	Day 5	Day 7	Day 10	Day 20+	Reference range
WBC (×10³/µL)	17.21	15.36	19.36	16.6	17.39	12-13	4.5-11.0
Platelets (×10³/µL)	143	146^*^	148	-	-	-	150-400
Lactate (mmol/L)	2.1	1.5	WNL	-	-	-	0.5-2.0
Troponin (ng/mL)	2.29	-	1.18	-	-	-	0.04
Creatinine (mg/dL)	Baseline 0.8-1.0	-	-	-	1.39	1.59-1.8	0.6-1.2
TSH (µIU/mL)	-	-	9.12	-	-	-	0.4-4.0
Vancomycin trough (µg/mL)	-	17.5	-	-	-	-	10-20

On hospital Day 27, the patient's code status was changed to do not resuscitate/do not intubate (DNR/DNI). Intravenous ampicillin 2 g every four hours and intravenous ceftriaxone 2 g every 12 hours were continued via a peripherally inserted central catheter (PICC) line. Oral amiodarone 200 mg twice daily was added for ventricular tachycardia.

While being repositioned in bed, the patient suddenly became unresponsive, with agonal breathing, an oxygen saturation of 74%, and a heart rate of 38 beats/min. The healthcare proxy was contacted, after which comfort measures were initiated. The patient died shortly afterward.

## Discussion

This case demonstrates how diagnostic delay, embolic complications, and a complex surgical timing dilemma converged fatally in a patient with *E. faecalis* aortic valve endocarditis. Although each complication is recognized individually, their simultaneous occurrence resulted in an insurmountable therapeutic challenge. Five key teaching points arise from this case.

The diagnostic pitfall of discordant blood and urine cultures

A key lesson from this case is how a concurrent *E. coli* UTI masked the true source of sepsis. On admission, blood cultures grew *E. faecalis*, while urine cultures grew *E. coli*. This discordance, identified early in hospitalization, should have indicated that the bacteremia was not urinary in origin and that another endovascular source, particularly IE, needed to be excluded. However, the initial UTI diagnosis was accepted as the sole explanation, delaying investigation for IE by several days.

The magnitude of this diagnostic gap is underscored by the landmark prospective study by Dahl et al., which systematically screened all patients with *E. faecalis* bacteremia using echocardiography and found an IE prevalence as high as 26%, far exceeding prior estimates of 5%-13% from retrospective studies with low echocardiography rates [4]. In a subsequent analysis, Dahl et al. argued that *E. faecalis* should be recognized as a “typical” endocarditis bacterium, equivalent to *S. aureus* and viridans group streptococci, demonstrating that this reclassification improved the sensitivity of the Duke criteria from 70% to 96% for identifying definite IE [5]. Dahl et al. identified six independent predictors of IE: community-acquired infection, unknown infection source, prosthetic heart valve, immunosuppression, monomicrobial infection, and ≥3 positive blood culture bottles. Our patient had at least two of these risk factors (community-acquired infection and monomicrobial *E. faecalis* bacteremia), placing him in the intermediate-risk category, with an IE prevalence of approximately 14% [4].

This case supports the consensus that all patients with *E. faecalis* bacteremia should receive echocardiographic screening for IE, even if a primary source appears evident. Clinicians should recognize that when blood and urine cultures yield different organisms, the source of bacteremia remains unexplained, and endocarditis must be actively ruled out.

Limitations of transthoracic echocardiography and the imperative for early transesophageal echocardiography

The initial TTE on hospital Day 2 showed preserved ejection fraction, no wall motion abnormalities, and no vegetations. This negative result may have provided false reassurance and contributed to a five-day delay before TEE was performed. During this time, the patient had persistently positive blood cultures and may have experienced additional septic emboli.

The limitations of TTE in detecting IE are well established. TTE has lower diagnostic accuracy than TEE, with a sensitivity of approximately 60% for TTE versus >90% for TEE in detecting vegetations [6]. This gap is even more pronounced for detecting perivalvular abscess, for which TEE sensitivity exceeds 85% with specificity >95%, compared with approximately 50% for TTE [6]. The 2015 American Heart Association (AHA) Scientific Statement specifically identifies morbid obesity as a condition that can preclude optimal echocardiographic windows and recommends that TEE be performed as soon as possible after TTE in such patients [7]. Our patient's morbid obesity (weight approximately 440 lb) was an additional factor that likely limited TTE image quality.

Furthermore, TEE may miss early paravalvular abscesses, particularly when performed early in the patient's illness, as an incipient abscess may appear only as nonspecific paravalvular thickening. Repeating TEE three to five days after an initial negative result is recommended when clinical suspicion for IE persists [7]. When clinical suspicion remains high after a negative TTE, TEE should be performed; if TEE is also negative, it should be repeated several days later.

In retrospect, *E. faecalis* bacteremia, persistently positive blood cultures despite appropriate antibiotics, and morbid obesity should have prompted TEE within 24-48 hours of the initial positive cultures rather than after five days. Proceeding directly to TEE is appropriate in high-risk cases, especially when suspicion for IE remains high, and echocardiography is inconclusive [6]. The opportunity for earlier TEE was missed between Day 2 and Day 4, with TEE delayed until Day 6.

The surgical timing dilemma: when indications and contraindications collide

This case presented a central therapeutic conflict: the patient had both Class I indications for urgent cardiac surgery and absolute contraindications to it. He had a perivalvular abscess at the aorto-mitral curtain, a 2.1 cm × 0.8 cm mobile aortic valve vegetation, severe aortic regurgitation with a flail left coronary cusp, persistent bacteremia for five days despite appropriate antibiotics, and progressive heart failure. Taken together, these findings support early surgery according to current guidelines.

However, the patient simultaneously had bilateral subacute cerebral infarctions with hemorrhagic transformation and SAH, placing him at high risk for perioperative neurological deterioration. The 2015 AHA Scientific Statement states that "in patients with major ischemic stroke or intracranial hemorrhage, it is reasonable to delay valve surgery for at least 4 weeks" (Class IIa, Level of Evidence B) [7]. This recommendation is supported by a multicenter study cited in the AHA Statement, which demonstrated that mortality was 75% when surgery was performed within four weeks of a hemorrhagic event, compared with 40% when surgery was delayed beyond four weeks [7]. The prohibitive perioperative risk of cardiac surgery within four weeks of a cerebrovascular event is driven by several converging pathophysiological mechanisms associated with cardiopulmonary bypass (CPB). First, CPB requires systemic anticoagulation with high-dose unfractionated heparin to prevent circuit thrombosis, which substantially increases the risk of hemorrhagic transformation of existing cerebral infarcts or new intracranial hemorrhage in the setting of a disrupted blood-brain barrier [8]. Second, CPB generates nonpulsatile blood flow that may impair cerebral autoregulation, potentially extending the ischemic penumbra surrounding a recent infarct [9]. Third, CPB induces acquired platelet dysfunction through multiple mechanisms: shear stress from the bypass circuit can mechanically damage platelets, contact with artificial surfaces can activate and consume coagulation factors, hemodilution can reduce platelet counts and fibrinogen concentrations, and hypothermia can further impair platelet aggregation [9]. Even after heparin reversal with protamine, platelet dysfunction may persist, compounding the hemorrhagic risk. Fourth, CPB triggers a systemic inflammatory response involving proinflammatory cytokines, including tumor necrosis factor-alpha (TNF-α), Interleukin (IL)-1, IL-6, and IL-8, which can increase capillary permeability; in the context of a recently infarcted brain with a compromised blood-brain barrier, this may further predispose to cerebral edema and hemorrhage [9]. After a hemorrhagic stroke, the risk of exacerbation by surgery is particularly high during the first month but may extend beyond one month in some patients, possibly because of undetected mycotic aneurysms [7].

A narrative review by Siquier-Padilla et al. in the Journal of Clinical Medicine confirmed that a four-week delay remains the recommendation for most cases of intracranial hemorrhage, although selected patients may undergo surgery earlier [10]. The Journal of the American College of Cardiology (JACC) Focus Seminar by Dayer et al. similarly noted that “if there has been an intracranial hemorrhage, a delay of 4 weeks is recommended, but this depends on the appearance and size of the cerebral lesion, the urgency of the cardiac indication, and the patient's general status” [11].

Our patient's STS risk score of 10% and six-month mortality risk of 67% further underscored the prohibitive operative risk. The patient was evaluated at three facilities with escalating levels of surgical capability, each declining to operate, a trajectory that, while clinically realistic, is rarely documented in the literature and highlights the systemic challenge of managing patients who fall into this therapeutic gap.

Prognostic implications of stroke in infective endocarditis

Stroke is one of the most devastating complications of IE and carries profound prognostic implications. IE is complicated by stroke in 20% to 40% of cases, and stroke is an independent adverse prognostic factor for survival [12]. The risk of stroke is highest at diagnosis and decreases rapidly after the initiation of antibiotic therapy, with the incidence dropping from 4.82 per 1,000 patient-days in the first week to 1.71 per 1,000 patient-days in the second week [12]. Systemic embolization occurs in 22% to 50% of cases of IE, and up to 65% of embolic events involve the central nervous system.

In the specific context of enterococcal IE, the International Collaboration on Endocarditis (ICE) prospective cohort study by Chirouze et al., the largest international series of enterococcal IE, reported a one-year mortality rate of 28.9% (144/500) and identified stroke as an independent predictor of one-year mortality (HR, 1.9; 95% CI, 1.3-2.8; p=0.001) [11]. Heart failure (HR, 2.4; 95% CI, 1.7-3.5) and age were the other independent predictors of mortality. Notably, surgery was not associated with better outcomes in this cohort [13].

A Danish nationwide propensity score-matched cohort study by Klein et al. demonstrated that the risk of ischemic stroke was highest during the first four weeks after IE diagnosis (HR, 57.20; 95% CI, 45.58-71.78) and remained moderately elevated for up to two years. This elevated risk was unrelated to atrial fibrillation or the presence of mechanical valves [14].

The combination of bilateral embolic strokes with hemorrhagic transformation, SAH, perivalvular abscess, severe aortic regurgitation with heart failure, and inability to undergo surgery placed our patient in a very high-risk prognostic category. The later development of refractory volume overload, AKI, and new-onset atrial fibrillation with ventricular tachycardia reflected the relentless progression of severe endocarditis without source control.

Discordant transesophageal echocardiography findings between institutions: an underappreciated challenge

A key finding in this case was the discordance between the initial TEE at the referring hospital, which showed a perivalvular abscess, a 2.1 cm × 0.8 cm vegetation, and a possible LAA thrombus, and the repeat TEE at the tertiary center, which showed a flail left coronary cusp with severe aortic regurgitation but no abscess or LAA thrombus. This discrepancy had significant clinical implications because the presence or absence of a perivalvular abscess is a Class I indication for surgery.

Several explanations exist for this discordance. First, true interval change is possible; the abscess may have partially resolved or ruptured with ongoing antibiotic therapy. Second, differences in imaging equipment, probe positioning, and operator experience may account for the discrepancy. Third, the 2020 American College of Cardiology (ACC)/AHA Guidelines acknowledge that TEE may miss initial paravalvular abscesses, particularly when performed early in the course of the patient's illness. In such cases, the incipient abscess may appear only as nonspecific paravalvular thickening, which, on repeat imaging over several days, may become recognizable as it expands and cavitates. Negative early TEE images do not exclude the potential for development of paravalvular complications [15]. Conversely, the initial TEE may have overinterpreted nonspecific findings.

This discordance highlights the importance of multimodality imaging in complex IE cases. The 2020 ACC/AHA Guidelines state that CT is useful for evaluating patients with equivocal TEE findings and for assessing complications in suspected paravalvular infection [15]. A landmark study by Feuchtner et al. in 37 patients with suspected IE showed that multislice CT detected all paravalvular abscesses and pseudoaneurysms, while TEE missed one case. CT also provided superior anatomic detail regarding perivalvular extent, including myocardial, pericardial, and coronary sinus involvement. Three-dimensional visualization of periannular tissue destruction and the relationships of perivalvular pseudoaneurysms was especially helpful for presurgical planning [16]. In this case, cardiac CT could have clarified the discrepancy between the two TEE studies.

Limitations

This case report has several limitations common to single-case observations. The exact timing of endocarditis onset relative to presentation is uncertain. The patient's two-week history of fever, malaise, and diarrhea before the initial ED visit may have been the first sign of IE rather than a separate gastroenteritis episode, although this remains speculative. The exact portal of entry for *E. faecalis* bacteremia could not be definitively established. Given that *E. faecalis* is a commensal organism of the gastrointestinal tract, the patient's two-week prodrome of diarrhea and fever may have represented GI mucosal disruption facilitating bacterial translocation, although this remains unproven. Notably, the urinary tract was effectively excluded as the source by the discordance between blood cultures (*E. faecalis*) and urine cultures (*E. coli*). Additionally, the diffuse lymphadenopathy seen on CT was not further investigated and may represent reactive changes due to systemic infection, although malignancy was not formally excluded.

## Conclusions

This case highlights three key lessons for clinicians managing *E. faecalis *bacteremia: (1) discordant blood and urine culture organisms should prompt immediate evaluation for endocarditis rather than attributing bacteremia to a urinary source; (2) TTE alone is insufficient to exclude IE, especially in obese patients, and TEE should be performed early when suspicion is high; and (3) the combination of surgical indications and neurological contraindications in IE with embolic stroke and hemorrhagic transformation creates a therapeutic impasse with a poor prognosis, emphasizing the need for earlier diagnosis to prevent this devastating cascade.

Furthermore, the management of IE complicated by acute ischemic or hemorrhagic stroke remains highly complex and demands a robust multidisciplinary approach, drawing on the coordinated expertise of vascular neurology, endovascular neuroradiology, neurosurgery, ID, cardiology, critical care, and cardiothoracic surgery. Given the specialized interventions required, timely transfer to a high-volume tertiary or quaternary center with a dedicated endocarditis team is essential to optimizing patient outcomes.
